# New Advances in Iberian Medieval Agriculture: Plant Remains from the Islamic Site of Castillo de Valtierra (Navarre, Northern Spain)

**DOI:** 10.3390/plants13213047

**Published:** 2024-10-31

**Authors:** Antonio Peralta-Gómez, Leonor Peña-Chocarro, Jesús Lorenzo Jiménez

**Affiliations:** 1 Departamento de Historia Medieval, Ciencias y Técnicas Historiográficas, Universidad de Granada, Campus de la Cartuja, C/Prof. Clavera, s/n, 18011 Beiro, Spain; 2 Laboratorio de Arqueobiología, Instituto de Historia (CCHS-CSIC), C/Albasanz 26-28, 28037 Madrid, Spain; leonor.chocarro@csic.es; 3 Departamento de Filología e Historia, Universidad del País Vasco/Euskal Herriko Unibertsitatea, P.º de la Universidad, 5, 01006 Vitoria-Gasteiz, Spain; jesus.lorenzo@ehu.eus

**Keywords:** agriculture, crops, Islamic, medieval Iberia

## Abstract

There has been a notable lack of archaeological research into the medieval period in Iberia, particularly in comparison to earlier periods. Consequently, the majority of our current understanding of agricultural practices and plant food sources in this region is derived from textual sources. However, there has been a notable increase in interest in archaeobotanical studies in medieval contexts over the past decade. In this context, this paper presents the results of a study of plant remains from Castillo de Valtierra (Navarre), with the objective of providing insights into agricultural practices and dietary habits during the Islamic period. In this area (the Ebro Valley), the Islamic period is divided as follows: Emiral period 756–929 AD, Caliphal period 929–ca.1012 AD, Taifal period ca.1012–1119 AD. This period was followed by the Christian period from 1119 AD onwards. Samples were collected from a variety of contexts in a systematic manner. A total of 2574 remains were recovered, and 57 taxa were identified. The findings of this study demonstrate that the community that inhabited Valtierra was primarily engaged in agricultural activities and had access to a diverse range of crops sourced from various productive areas, including cereal fields, home gardens, and forests.

## 1. Introduction

The subject of plant diet in Medieval Islamic Iberia is one that has yet to be fully explored. While there is a plethora of information available from textual sources regarding agricultural practices and crops, there is considerably less data concerning the actual remains of agricultural products and, more generally, plant remains. This is largely due to the limited attention that medieval Iberian archaeologists have dedicated to this area of research and the prehistoric focus that has been maintained by the majority of archaeobotanists working in Iberia. For at least the last 30 years, there have been significant developments in the field of medieval archaeology in Iberia. A considerable number of sites have been excavated, yielding a vast quantity of archaeological material, and a wealth of information [1]. Nevertheless, archaeobotany has remained an overlooked area of research in these developments, and strategic sampling and flotation techniques have been rarely applied. This situation is in stark contrast with the developments that have occurred in other European countries, where medieval archaeobotany has flourished resulting in the generation of a substantial corpus of data [2,3,4,5,6,7,8,9,10,11,12,13,14]. In recent years, there has been a notable increase in the number of researchers working on the medieval period in the Iberian peninsula resulting in a steady output of publications. These include comprehensive overviews that provide updated bibliographic information on Iberian studies [15,16,17] and detailed reports on Christian [18,19,20] and Islamic sites [21,22,23,24,25,26]. Furthermore, recent years have seen a significant expansion in the scope of research in the field of Islamic archaeology, with the introduction of new topics and innovative methodological approaches. Following initial research into hydraulic systems [27,28,29], which greatly enhanced our understanding of agricultural practices, researchers are now exploring new areas of research such as archaeobotany, archaeozoology and novel methodologies, including the study of stable isotope analyses [30]. These insights are providing valuable data on the exploitation of plant (see references above) and animal resources [31,32] as well as on the management of different productive spaces. The wealth of the discipline is also reflected in the number of recent projects that have been initiated.

The topic of Islamic agriculture has been the subject of considerable research, with a consensus emerging that the expansion of Islam was responsible for a significant transformation of agricultural practices. This conclusion has been supported by a combination of archaeological evidence and textual sources. In 1974, A.J. Watson published his seminal work The Arab agricultural revolution [33], which was followed by a second publication in 1983, entitled Agricultural innovation in the Early Islamic world: The diffusion of crops and farming techniques, 700–1100 [34]. These two works, based on the available written sources at the time [35,36], established the premise for the pervasive notion of a “medieval green agricultural revolution”, which occurred between the 7th and the 11th centuries AD, following the unprecedented unification of a vast region extending from Central Asia to the Iberian Peninsula. This significant undertaking would have facilitated the movement of people and the dissemination of ideas, techniques such as irrigation, and new crops. Watson identified 18 sub-tropical species, including aubergine, spinach, sugarcane, citrus species, rice, hard wheat, cotton and others, which were likely spread across the region. Several authors have criticized the model [37] arguing that some of the species were already in use prior to the arrival of the Arabs. However, as Squatriti [38] has rightly pointed out the role of Islamic communities in the diffusion and translocation of these species to new regions, and their subsequent acclimatisation to different environments, is beyond question. Forste et al. [36] has correctly asserted that Watson’s model is best understood as a synthesis of the historical evidence regarding crop introductions and farming methods available at the time. Further research based on direct evidence of agriculture will undoubtedly facilitate a more comprehensive understanding of the specific crops, their introduction, and the timing of such introductions during the Islamic period.

In this context we present the archaeobotanical study of Castillo de Valtierra (Navarre), a paper that examines agriculture and diet during the Islamic period using data from the systematic recovery of plant remains. The objective is to identify the components of arable farming, the productive spaces in which they were cultivated, and to explore the potential uses of these species in order to gain insight into the plant diet of the community.

## 2. The Site

**Figure 1:**
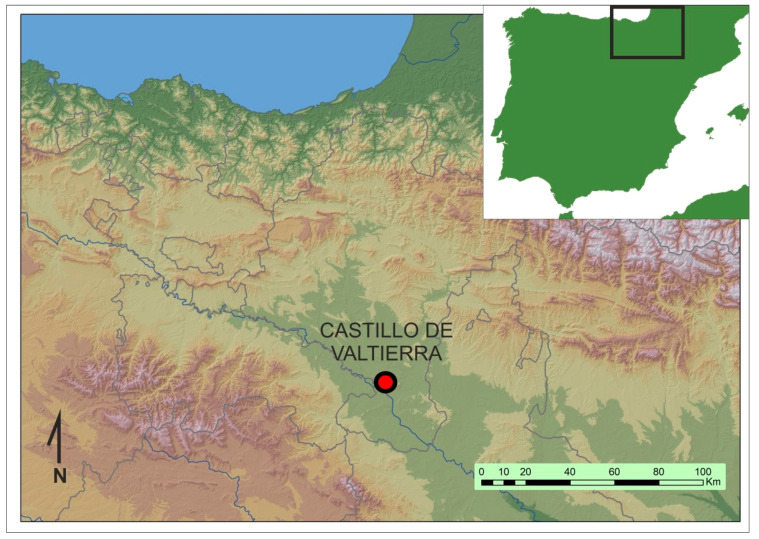
Location of the site.

The town of Valtierra is situated on the left bank of the Ebro River, within the alluvial plain formed by the river at its confluence with the Aragón River, which is currently located within the autonomous community of Navarre, north of Spain (Figure 1). Due to a series of historical events, this location held a border position throughout the Middle Ages. Initially, it was situated between the Arab-Islamic and Latin-Christian worlds. Subsequently, following its conquest by Aragon in 1119, it became a border between the kingdoms of Castile and Navarre.

The settlement’s history prior to the Islamic conquest in 711 is largely undocumented. However, its proximity to the Islamic *madīna* of Tudela, established in 802, suggests the possibility of a close connection between the two locations. The earliest known references to the site date back to the turbulent period of the late 9th century, when it became a bargaining chip between the various Islamic powers in the area, embroiled in constant internal conflicts. According to the Arabic chronicles, Sancius, King of Pamplona, conducted a raid on the castle of Valtierra in 918, resulting in the destruction of the mosque. This attack prompted a response from the Emir of Córdoba, a young Abd al-Rahman III, who initiated a punitive campaign against the Kingdom of Pamplona, thereby restoring Islamic authority in the region.

Following the proclamation of the Caliphate of Córdoba in 929, Valtierra is no longer referenced in the sources. It reappears at the end of the 11th century in a markedly different context, characterised by ongoing turbulence. This was a time of persistent pressure from the Latin kingdoms of the northern peninsula. A document dated 1093 makes reference to the existence of a church in Valtierra, which was still under Islamic rule at the time. This provides evidence of the continued presence of a Christian community in the area. The conquest of the *taifa* of Zaragoza occurred in 1119, resulting in the fall of Valtierra into the hands of the Christian Kingdom of Aragon.

The transfer of power from the Aragonese to the Navarrese monarchy in 1134 resulted in the southern border of the Kingdom of Navarre being defined as the limits of the Ebro Valley, encompassing the city of Tudela. Valtierra thus became a frontier enclave, facing Aragon and, in particular, Castile, which from the outset demonstrated its annexationist ambitions. This would have resulted in the construction of a castle in the area, as evidenced by written documentation. It seems probable that the population was relocated to the area currently occupied by Valtierra, with only a fortified space at the top of the hill remaining, as referenced in the documentation. In conclusion, Valtierra and the entirety of the Kingdom of Navarre were ultimately subjugated by Castile following the 1512 conquest led by Ferdinand the Catholic.

**Figure 2:**
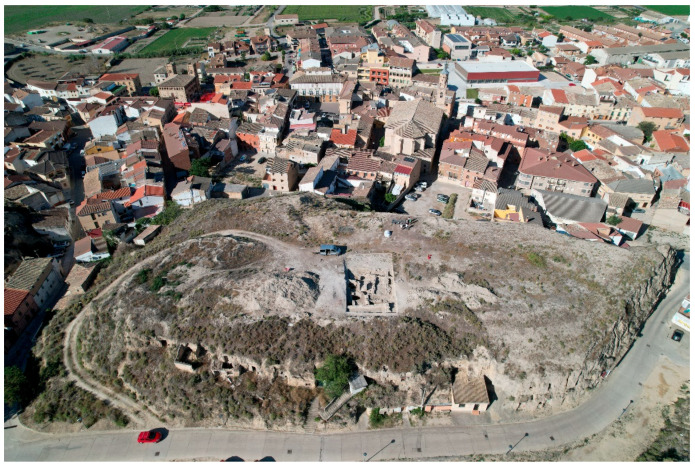
Aerial view of the site on a hilltop. Photo: J. Lorenzo Jiménez.

The archaeological site of El Castillo de Valtierra is situated on a flat-topped hill that rises above the urban core of present-day village of Valtierra (Figure 2). In 2019, an initial georadar survey was conducted, which revealed the presence of subsurface structures. This led to the start of excavations the following year. The area currently undergoing excavation covers 15 × 20 metres, representing just 5% of the total area of the hill on which the site is located.

The 2023 excavation has revealed the existence of an Islamic settlement dating back at least to the 9th century AD (Phase 1), followed by several restructurings, all within the Islamic period, between the 10th century and the early 12th century AD (Phases IIa-b), and a later phase (Phase III) from the beginning of the 12th century which corresponds to the Christian occupation (Table 1). It is worth noting that the excavated area shows evidence of a burnt layer covering a significant portion of it which separates Phase I and Phase II where different structures (spaces) have been identified. The superimposed layers above these structures from the post-Christian conquest period are evident, discernible through ceramics but also through a different building style. These structures preceded the total abandonment of the hill at an undetermined time after the 16th century.

Phase I encompasses the earliest levels of spaces 4, 5, 6, and 7, as well as those of spaces 2 and 3, which were originally a single room. On top of these older levels, the aforementioned burnt layer was documented, with a greater intensity in space 2, although it extended throughout the entire excavated area, followed by a thin levelling fill. During Phase II, new walls were constructed over the burnt layer, specifically EC 23.53, which divided the original space into spaces 2 and 3, and EC 20.8, which cuts through wall EC 21.11 and divided space 5 into two different units.

**Figure 3:**
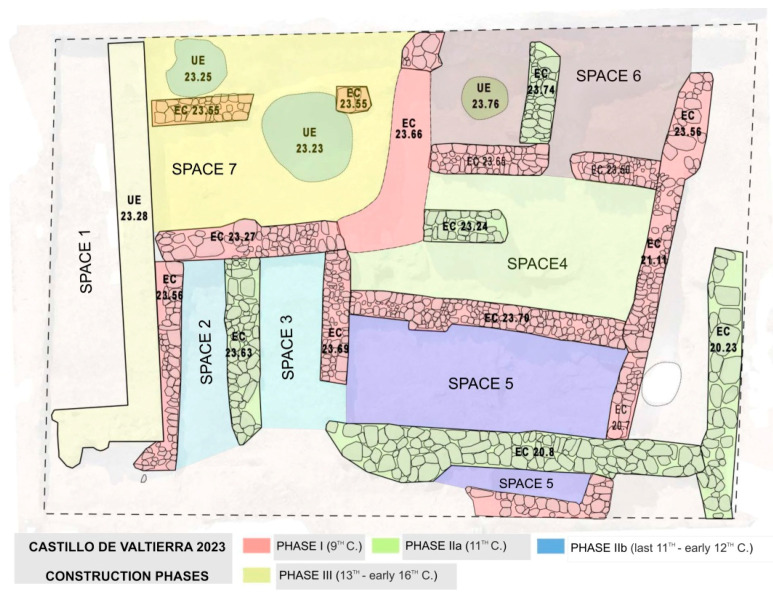
Plan showing the various structures of the site designed by the directors of the excavation.

All the identified spaces were assigned numbers, as illustrated in Figure 3.

## 3. Materials and Methods

A total of 16 samples were taken from various structures within the excavation area during the 2023 campaign, representing the three different phases (Table 1). The sampled contexts comprise fills from a variety of sources, including foundation trenches, pits, potential drains and ashy fills. The sample size ranges from 33 to 18.5 litres of sediment, while the flot volume oscillates between 1450 and 40 mL (Table 2).

All soil samples were transported to the Laboratorio de Arqueobiología of the Spanish National Research Council (Instituto de Historia) in Madrid, where they were processed in a flotation machine fitted with a 1 mm mesh inside the flotation tank and 0.25 mm outside. Subsequently, the flots were sieved in a series of columns with progressively smaller mesh sizes (4, 2, 1, 0.5 and 0.25 mm) to facilitate the sorting process. Each fraction was then sorted using a Leica stereomicroscope (×50) with seeds, fruits and other botanical elements picked out.

With the exception of the sample from UE 23058, which was subsampled due to the considerable quantity of remains, all samples were fully sorted. The figures presented in the table from UE 23058 correspond to the actual number of remains found in 100 mL of sediment.

The remains were identified using the reference collection of the Laboratorio de Arqueobiología as well as a number of seed atlases [39,40,41]. Botanical names of cultivated plants follow the traditional binomial classification used by Zohary et al. [42], whereas the nomenclature of wild specimens adheres to the Flora Iberica [43].

In this study, entire fruits/seeds, as well as fragments with visible embryos were counted as single units.

It is worth noting that, while charring is the most prevalent method of preservation in the Iberian Peninsula, the plant material from Castillo de Valtierra has been preserved in either a charred or mineralized state. Once the charring process is complete, provided it has not been overly aggressive, the plant remains are preserved in their original morphology, allowing for greater taxonomic detail to be identified. In the case of mineralisation, the process occurs when the remains come into contact with calcium carbonate or silica, which fills the remains’ cells, creating a replica of the original remains.

## 4. Results

The samples analyzed have yielded 2574 remains, including 16 cultivated species. Table 2 provides a comprehensive overview of the results, including details on the stratigraphic unit (UE) and soil and flot volumes. The number of items per sample varies considerably, from a low of just a few (between 2 and 12) in UEs 23.020, 23.077, 23.078, 23.43, 23.026 and 23.036, to a high of dozens, hundreds and thousands in the remaining samples.

**Figure 4:**
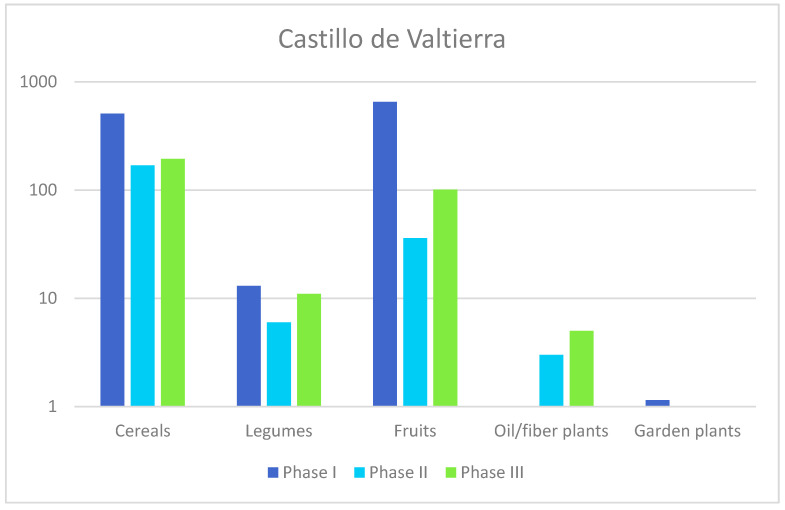
Comparative analysis of different categories of edible plants across the three identified phases.

Apart from the presence or absence of certain species, such as coriander (*Coriandrum sativum*), which is only documented in 9th-century levels (Phase I), no temporal differences are observed (Figure 4). There are, however, contrasts between contexts in terms of the number of remains and the diversity of the sample. The richest samples in terms of plant variety have been recovered from Space 5 (UE 23.058 and UE 23.073), from a ashy layer dated to Phase I and Space 7, from a courtyard dated to Phase II (UE 23.014), and from a pit excavated in Space 7 dated to Phase III (UE 23.040 and 23.032) (Table 1). In comparison, the remaining samples corresponding to fills of different features are less rich. Sample from UE 23.053 derived from a drain has yielded a significant quantity of mineralized fig (*Ficus carica*) and grape pips (*Vitis vinifera*).

**Figure 5:**
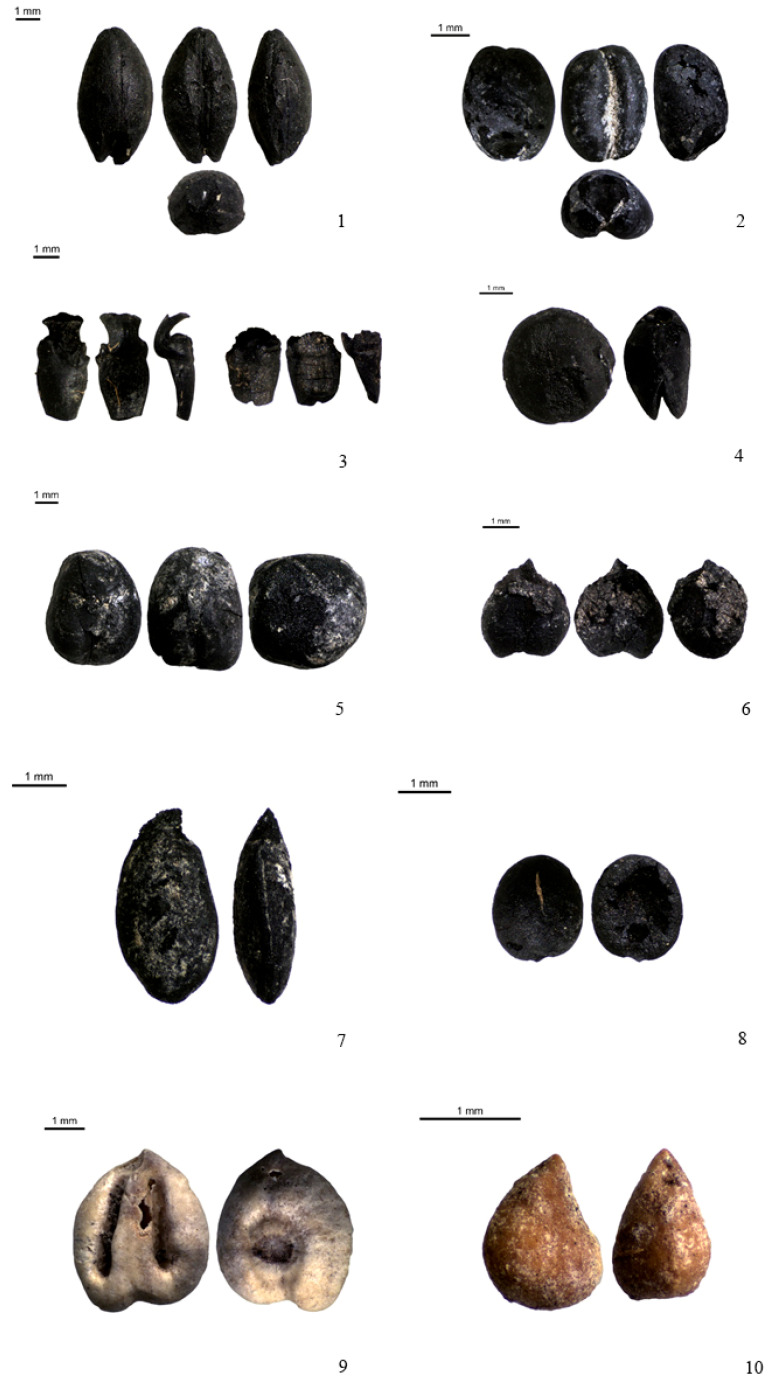
Some of the main plant remains found at Valtierra. 1. *Hordeum vulgare* ssp. *vulgare*; 2. *Triticum aestivum/durum*; 3. *T. aestivum* and *T. durum* rachis; 4. *Lens culinaris*; 5. *Vicia faba*; 6. *Cicer arietinum*; 7. *Linum usitatissimum*; 8. *Coriandrum sativum*; 9. *Vitis vinifera*; 10. *Ficus carica*. Scale: 1 mm.

The cultivated species are primarily represented by cereals, legumes, fruit trees and oil plants (Figure 5). Five different cereals have been identified, namely hulled barley (*Hordeum vulgare* ssp. *vulgare*), bread wheat (*Triticum aestivum*), durum wheat (*Triticum durum*), common millet (*Panicum miliaceum*) and oat (*Avena sativa/strigosa*). Naked wheat (*T. aestivum/durum*) is by far the most abundant cereal, followed by barley and oat.

In addition to the caryopsis, a significant number of cereal chaff remains has also been retrieved. This has enabled the identification of both hexaploid wheat and tetraploid wheat. Furthermore, a considerable quantity of barley chaff has been discovered, whereas only two floret bases of oat have been identified. In line with Jacomet [39] the remains have been identified as cultivated oat, but it has not been possible to distinguish between *A. sativa* and *A. strigosa*.

Legumes are represented by a great diversity of species, including lentil (*Lens culinaris*), fava bean (*Vicia faba*), grass pea (*Lathyrus sativus*), bitter vetch (*Vicia ervilia*) and chickpea (*Cicer arietinum*), which are represented by just a few specimens. Pea is absent from the archaeobotanical record.

The main species of fruit represented among the samples are grapes (*Vitis vinifera*) and figs (*Ficus carica*), both of which have been preserved by charring and mineralization. Whole fruits, pips and pedicels from grapes have been identified. Other fruit species present at the site include sloe (*Prunus spinosa*), walnut (*Juglans regia*), blackberry (*Rubus* sp.), raspberry (*Rubus idaeus*) and rowan (*Sorbus* sp.).

This study has also yielded remains of other cultivated plants such as flax (*Linum usitatissimum*), which is represented not only by seeds but also by a capsule with seeds inside. Additionally, a fragment of a single seed of coriander (*Coriandrum sativum*) has been identified.

A total of 44 taxa belonging to 16 families have been identified among the wild plants. The most prevalent families are Poaceae, Rosaceae, Amaranthaceae and Rubiaceae. Many of the identified species are weeds of crops, while others correspond to groups of plants growing in and around the site.

## 5. Discussion

The rich archaeobotanical assemblage from the Islamic contexts at Valtierra provides a fascinating insight into the agricultural economy of the site from the 9th to the 12th and later centuries AD.

**The cereals**

The archaeobotanical study has demonstrated that this community cultivated a diverse range of cereals. Farming was undoubtedly the primary occupation of the people of Valtierra, with cereals representing a significant source of calories in their diet. Among the cereals, free-threshing wheat (both bread wheat and durum wheat) (*Triticum aestivum/durum*) and hulled barley (*Hordeum vulgare* ssp. *vulgare*) are the most important. Additionally, oats (*A. sativa/strigosa*) are also present, while millets (common millet –*Panicum miliaceum*- and foxtail millet –*Setaria italica*-) are scarcely represented. These are common crops that have also been identified in other contemporary sites whether Islamic or Christian [16,19,20,21,24,26], and they represent the most common cereal species in Iberia since prehistoric times.

Wheat is represented by two free-threshing species (*T. aestivum* and *T. durum*) from which both caryopses and rachis have been recovered in large numbers, particularly from Space 2, 5 and 7. Both wheat species are usually grown in dry fields together with barley, although historical sources also attest to the cultivation of irrigated cereals. This is also the case of barley which has been identified as 6-row hulled barley *(H. vulgare* ssp. *vulgare*), representing the main barley species in Iberia since at least the Bronze Age. In terms of ecological requirements, barley displays greater tolerance to drought and frost than wheat and so it is better adapted to grow in adverse environmental conditions and poor soils where it provides higher return rates than other cereals.

For the period and region under study (Al-Andalus), there are a number of texts written by Hispano-Arab agronomists, that provide a comprehensive insight into a range of subjects, including crop diversity, agricultural practices as well as on animal husbandry, medicinal plants, storage and food preparation. According to these sources, wheat and barley were used in a multitude of ways. The grains were crushed or ground into flour to make bread and different types of pastries, or cooked into soups, pottages, porridge and gruel to prepare a variety of dishes. These could be mixed with water, milk, juice, honey, oil, vinegar or animal fat, seasoned with herbs and spices and consumed with meat, nuts, vegetables, fruit, etc. Bolens [44] includes several recipes in which wheat is one of the main ingredients.

Despite barley being typically regarded as a cereal primarily utilized for animal feed, archaeological [45] and documentary evidence indicates that it was also consumed by humans. Hulled barley is the most common type of barley in this period. Caryopses and chaff have been identified in almost all samples and in some cases in large quantities. In the 12th century AD, Avenzoar, a renowned Arab, physician, wrote his Kitab al-Aghdiya, a comprehensive manual on foods and nutrition. The work includes descriptions of the various types of bread, which encompass barley bread as well as other preparations with this cereal [46]. Moreover, data from pre-colonial Morocco [47] provide a comprehensive account of the common use of a preparation consisting of mixing roasted barley or wheat flour with water. This appears to have been a common peasant meal.

Other cereals present in Valtierra (in low quantities) include the millets, namely common millet (*P. miliaceum*) and foxtail millet (*S. italica*), which have been identified in Space 7 on top of a courtyard floor (Phase II). Both are spring cereals, characterized by a short growing cycle. The sowing of the seeds occurs in the spring, with the harvesting taking place during the summer. It has traditionally been assumed that the presence of millets in the archaeological record, together with other cereals, could be associated with a double annual harvest. However, Teira-Brión [48] indicates that this assertion cannot be upheld, as the decision to cultivate a specific crop depends on a variety of factors, including agricultural systems, social organization, environmental conditions and, ultimately, farmers’ decisions as suggested by other authors [49].

Common millet and foxtail millet are resilient cereals that are well adapted to a range of environmental conditions, making them ideal for a variety of agricultural settings. They can be cultivated in poor soils in dry areas. It is clear from written sources that millets were consumed either as grains or ground into flour, which was used to produce bread or soups. Furthermore, ethnographic data [48,50] suggests that millets were also a valuable source of animal feed.

Further analysis has revealed the presence of oats. The majority of the remains are caryopsis, but the species could not be determined. The only chaff remains that have been preserved are of the cultivated type, but it has not been possible to distinguish between *A. sativa* and *A. strigosa*. Oats have a long history of use as a food source for humans and animals.

The high concentration of rachis remains from both wheat species but also from barley indicates that at some point during the agricultural process, these species were probably processed at the site. Following Hillman’s cereal processing sequence [51,52,53], the by-product of fine sieving, designated as the fine-cleanings, is a common occurrence within the archaeological record. The cleaning of grain from contaminants (such as cereal straw, chaff, weed seeds, etc.) requires the use of sieves of of varying dimensions and exact movements enabling the transfer of impurities to the surface of the semi-clean grain. In both cases, the contaminants include chaff remains (cereal rachis, awns) as well as weed seeds of varying dimensions, contingent on the sieve employed. Such cereal waste can be used for animal feed, lighting fire and other purposes. Sieving occurs at various stages of the crop processing sequence, typically just before the grain is stored or on a piecemeal basis.

In light of the considerable quantity of chaff remains in comparison with caryopses and the high number of plants that can be classified as arable weeds, it is plausible to hypothesise that a portion of the plant material may originate from mixed refuse deposits.

**The legumes**

Despite legumes are often misrepresented in the archaeological record, the Valtierra assemblage offers a large diversity of species. The number of specimens is limited, but the remains of fava beans (*Vicia faba*), lentils *(Lens culinaris*), grass pea (*Lathyrus sativus*), bitter vetch (*Vicia ervilia*) and chickpea (*Cicer arietinum*) have been successfully identified in different samples. All of them are well documented in the archaeological record of Iberia during the Middle Ages. Furthermore, they have been identified in other Islamic sites, as referenced in the literature [16,21,22,26,54]. Pulses could be cultivated in both dry fields and under irrigation in home gardens. In addition to their use as food for humans and animals, legumes have historically been employed as a means of restoring soil fertility. The ethnographic data [55,56] and written sources [46,57] for the period provide insights into the diverse ways of consuming pulses. They could be eaten dry in stews, fresh or ground into flour. Of the list of species identified, bitter vetch was probably used as animal feed, although there are records of its consumption by humans during periods of scarcity. Furthermore, lucerne (*Medicago sativa*) has been included among the cultivated pulses, given its long-standing role as a fodder plant. However, it is acknowledged that it may have been grown wild around the site.

**The fruits**

The archaeobotanical record from Valtierra has revealed the presence of two cultivated species, the grape (*Vitis vinifera*) and the fig (*Ficus carica*), which have been preserved mainly by charring but there are also mineralized remains. The majority of the mineralized fig remains were recovered from a context that has been interpreted as a drain, while the mineralized grape pips (only two) were retrieved from a large pit fill. The charred seeds come from different contexts. As is the case with other Islamic sites, these two fruit species are abundant reflecting their importance in the community’s diet. Both species were widely consumed by Islamic communities and throughout Medieval Iberia. A review of agronomic written sources reveals a wealth of information regarding the cultivation methods, use of irrigation and manure, and the various varieties of both species. Grapes and figs were consumed in either fresh or dried form. Furthermore, grapes could be consumed as must or used to produce vinegar. Both fruits were employed in a multitude of culinary preparations, including the production of sweets and syrups [44,58]. It is evident that the list of fruit trees used by Islamic communities was considerably more extensive. Some of these absent fruits have been identified at other contemporary sites, including the pomegranate (*Punica granatum*), sweet and sour cherry (*Prunus avium/cerasus*), plum (*Prunus domestica*), peach (*Prunus persica*), apricot *(Prunus armeniaca*), apple (*Malus domestica*), olive (*Olea europaea*) [16], the citrus fruits (*Citrus* spp.), quince (*Cydonia oblonga*), medlar (*Mespilus germanica*) [18], and the carob (*Ceratonia siliqua*) (unpublished data).

Others, however, are only recorded in written sources such as the watermelon (*Citrullus lanatus*), date (*Phoenix dactylifera*), mulberry (*Morus alba*), myrtle (*Myrtus communis*) or specific citrus species, including the orange (*Citrus sinensis*), the lemon (*Citrus limon*) or the citron fruit (*Citrus medica*) among others. Many of these fruit trees were probably cultivated in orchards under irrigation, although there is no evidence for this in the area.

Furthermore, Valtierra has furnished invaluable insights into the harvesting of wild fruit species. Indeed, the archaeobotanical record reveals the presence of numerous species, including the walnut (*Juglans regia*), sloe (*Prunus spinosa*), black mulberry (*Morus nigra*), raspberry (*Rubus idaeus*), and rowan (*Sorbus* spp.), which were likely gathered in the vicinity of the site. It is also possible that walnut was cultivated in orchards, although the lack of further evidence precludes further investigation. These fruits are thought to have constituted a supplementary element of the diet. Other species that are commonly found in other Islamic sites, such as almonds, pine nuts, and acorns, are notably absent from Valtierra.

**Garden plants**

In addition to the crops cultivated in fields and the exploitation of the natural environment, it seems plausible that small family irrigated gardens existed to provide vegetables, tubers and other garden plants to the members of the community. The archaeological evidence of garden plants is inherently limited by the nature of the preservation of the remains, which is subject to a number of constraints. Charring tends to destroy small and fragile remains, whereas waterlogging is considered to be the optimal preservation mode for garden plants. A previous study by one of the authors [15] analyzed the archaeobotanical evidence of horticultural plants in the Andalusi context. Despite the restricted nature of the evidence, several species were successfully identified in Islamic sites. A number of different plant species were identified, including celery (*Apium graveolens*), coriander (*Coriandrum sativum*), fennel (*Foeniculum vulgare*), mint (*Mentha* ssp.), poppy (*Papaver somniferum*) and members of the cabbage family (*Brassica nigra*), as well as carrot (*Daucus carota*). Additionally, the presence of garlic (*Allium sativum*) has also been confirmed [26].

Similarly, the data from Valtierra are scarce, yet a fragment of the inner part of a coriander seed (*Coriandrum sativum*) has been successfully identified. This species has been previously identified in other Islamic sites in Iberia, including Madinat Al-Zahara (Córdoba) with a chronology of the 10th century [16], Albalat (Extremadura) between the 10th–12th century AD [24], and in Portugal [59,60]. The precise dating for this latter example is not available. Coriander is arguably one of the most frequently utilized spices whether in its fresh form or as seeds. Avenzoar’s writings provide evidence of its use in a range of culinary preparations, including those based on meat, fish and eggs [46]. Furthermore, he recommends its use for oral hygiene.

Some of the identified families among the wild species, such as Apiaceae, Amaranthaceae and Brassicaceae, include other potential garden plants, but identification to species-level has not been possible. Written sources describe the cultivation of many of these plants in kitchen gardens, including artichokes (*Cynara cardunculus*), aubergines (*Solanum melongena*), cabbages (*Brassica* spp.), leeks (*Allium porrum*), onions (*Allium cepa*), and a plethora of other vegetables, as well as a multitude of aromatic plants. Nevertheless, the archaeobotanical record from Valtierra has not yielded any evidence in this regard.

Additionally, the archaeological record indicates the presence of several seeds and a capsule of flax (*Linum usitatissimum*), from a context dated to the 11th century AD. Furthermore, seeds were retrieved from a pit excavated in Space 7, with a chronology that postdates the 12th century AD. It seems probable that flax was cultivated in irrigated areas for fibre production, although flax can also grow in drylands. The presence of seeds may be indicative of cultivation for seed, or alternatively, may simply indicate seed storage for subsequent sowing. There is no doubt that flax was a significant fibre in the Islamic period. This is evidenced by the numerous references to it in agricultural books [61], which describe its cultivation and subsequent processing for the production of fibres.

**The wild plants**

Additionally, data has been retrieved from wild plants, with some specimens clearly representing weed seeds, e.g., the Rubiaceae (*Asperula arvensis*, *Galium* sp., *Sherardia arvensis*), Poaceae (*Lolium* spp., *Festuca* spp., *Poa* sp., *Phalaris* sp. etc.). Others can be considered ruderals growing in the site (Table 2).

**Arable farming in Valtierra**

Agriculture was central to the medieval communities, particularly in the rural areas. Cereals played a significant role in the diet of the population, providing a basic source of nutrition. Other than cereals, people in Valtierra also consumed legumes, fruits, horticultural and oily plants as well as wild plants gathered from the natural environment. These were obtained in a variety of productive spaces, including cereal fields, gardens, and forests. Cereals and legumes were probably cultivated on a rotation basis, although some legumes may also have been grown in small gardens for the production of green seeds (for example, peas).

One of the most significant issues pertaining to Islamic communities is the practice of irrigation, which has been extensively studied in the context of medieval archaeology in Iberia [28,29]. This topic has occupied a central position in the history of research. However, over the past few years, research conducted in Iberian dry land areas [62,63] has demonstrated that rain-fed agriculture was a common practice in large parts of the Iberian peninsula where irrigation was not feasible. The evidence provided by the cereal weed seeds from Valtierra, comprising mainly members of the grass family but also species of the Rubiaceae family (*Galium* sp., *Sherardia arvensis*, *Asperula arvensis*), which are typically considered arable weeds, does not provide evidence that cereals were irrigated. Additionally, no evidence of hydraulic infrastructure has been identified at Valtierra. This, of course, does not invalidate the practice of small-scale irrigation in kitchen gardens, which would provide leafy and root vegetables, herbs, bulbs, seeds and fruits used for food, medicines, dyes, crafts as well as for many other purposes. There is evidence to suggest that such practices existed in Valtierra, despite the limited evidence available.

Furthermore, information is also missing concerning the presence of crops traditionally associated with Islamic agriculture, including aubergine, spinach, citrus fruits, rice, and others. These crops were identified by Watson as a central element of his “Agricultural Green Revolution” theory. Further research and the analysis of additional samples from future excavations will be required to determine whether these crops were part of the Valtierra agricultural production or absent from the diet of the community.

## 6. Conclusions

The study of plant remains from Valtierra indicates that this community had access to a diverse range of crops, as evidenced by the presence of numerous species within the archaeological record. The dominant crops were free-threshing wheats and hulled barley. Millets and oats were also grown. A variety of legumes were cultivated alongside the cereals, including lentil, chickpea, fava bean, bitter vetch and grass pea. Bitter vetch may have been used for animal feed in addition to lucerne. Furthermore, farmers cultivated flax, fruits, and potentially horticultural products. The only evidence for the latter is a single coriander seed. Additionally, plants were gathered from the wild. There is currently no evidence of the presence of exotic crops that would support Watson’s model in the Valtierra area. The reasons for the presence of exotic plants are likely to be the result of a combination of factors, although taphonomic factors may have played an important role. Charring tends to preserve cereals and legumes, while many vegetables, including exotic species, are much less likely to survive. It is also important to consider that Valtierra is a rural settlement, which suggests that the introduction of exotic products, which are documented in Iberia from the 11th century onwards, may have occurred at a slower rate. The assemblage studied illustrates the exploitation of a range of productive areas, including fields in the drylands, vineyards, and potentially irrigated home-gardens associated with the cultivation of garden plants and fruit trees. The available data indicate that this pattern is present in both contemporary rural sites, whether Christian or Islamic.

The archaeobotanical evidence has contributed to explore the economy of Valtierra providing interesting insights into the economy of a rural Islamic site in Iberia. Our findings are consistent with those of other studies conducted at similar sites in Iberia. Further research will definitively contribute to advancing our understanding of the role of plants in the economy of Islamic communities, which remains a relatively understudied area in Iberia.

## Figures and Tables

**Table 1:** Distribution of samples according to site phasing with information on location, context and number of remains for each sample.

Castillo de Valtierra																
	Phase I (9th-First Half 10th AD) Emiral-Caliphal			Phase IIA (Second Half 10th-First Half 11th AD) Caliphal-Taifal					Phase IIB (Late 11th-Early–12th AD) Taifal	Phase III (Half 12th–16th AD) Christian						
UE	23.058	23.073	23.078	23.014	23.030	23.057	23.075	23.077	23.020	23.022	23.026	23.032	23.036	23.040	23.043	23.053
Location	Space 2	Space 5	Space 5	Space 7	Space 7	Space 4	Space 6	Space 6	Space 7	Space 7	Space 7	Space 7	Space 7	Space 7	Space 7	Space 7
Context	burnt layer	burnt layer	floor	patio floor	wall collapse	room fill	pit fill	pit fill	fill	pit fill	pit fill	pit fill	pit fill	pit fill	foundation trench	drain
Nº of remains	503	1053	8	202	58	66	30	12	2	53	7	155	12	205	4	75

**Table 2:** List of cultivated and wild plants (inserted at the end of the paper).

CASTILLO DE VALTIERRA	Space 2	Space 5	Space 5	Space 7	Space 7	Space 4	Space 6	Space 6	Space 7	Space 7	Space 7	Space 7	Space 7	Space 7	Space 7	Space 7
UE	23.058	23.073	23.078	23.014	23.030	23.057	23.075	23.077	23.020	23.022	23.026	23.032	23.036	23.040	23.043	23.053
SEDIMENT VOL. (L.)	24	22	17	26	33	18,5	22	17	21	25	19	21	26	23	19	19
FLOT VOL. (mL.)	600	500	70	150	500	250	100	90	100	350	40	200	1.450	700	100	150
***CEREALS***																
*Hordeum vulgare vulgare* (straight)	16	11		8		2	2					5		13		
*Hordeum vulgare vulgare* (twisted)	24	16	2	25	12	11	2	2		2		15	2	27		1
*Hordeum vulgare* (undec)	16	19		12		6	1	6	1	2		3	2	9		
*Hordeum vulgare* rachis	25	78		15	1					3		72				
*Hordeum vulgare* (glume base)		1														
*Triticum durum/aestivum*	227	61	1	25	12	17	2	3		9		14	3	40	1	
*Triticum aestivum* rachis	72	40		3	3							3				
*Triticum durum rachis*	2	4														
*Triticum* sp.	90	7		1	2		1			3	2			13		
*Panicum miliaceum*				2												
*Setaria* italica				1												
*Avena sativa/strigosa*		8			2							1				
*Avena* sp.	1	1										1				
*Avena sativa/strigosa* (floret base)												2				
Cereal indet.	2	7		3	1	6	2		1	1	1	7	4	13		
Cereal rachis							2									
***LEGUMES***																
*Cicer arietinum*										1						
*Lathyrus sativus/cicera*		3								2						
*Lathyrus* cotyledon				1				1								
Cf. *Lathyrus*										1						
*Lens culinaris*				3						4		1				
*Vicia ervilia*	1									1						
*Vicia faba*		1														
*Vicia faba* frag.										1						
*Vicia/Lathyrus*		2														
*Medicago sativa*		6		1												
***FRUITS***																
*Ficus carica* (seeds + fruit)		17														
*Ficus carica* (seed)	3	509	1	4	2	2	3									
*Ficus carica*(seed mineralized)			1		8										1	58
*Vitis vinifera* (fruit)		1												1		
*Vitis vinifera* (seed)		38	1	1	5	2	3			1				8		15
*Vitis vinifera* (mineralized)											1		1			
*Vitis vinifera* (pedicel)		1		1		1	1							13	1	
*Juglans regia* (pericarp frag.)										1						
*Prunus spinosa* (seed)		6														
*Rubus* sp. (seed)		38														
*Rubus idaeus* (seed)		34														
*Rubus idaeus/fruticosus*(seed)				3												
*Rubus* sp. (seed)	1															
*Sorbus* sp. (seed)		1														
***OIL/FIBER PLANTS***																
*Linum usitatissimum* (seed)				2	1					2				2		
*Linum usitatissimum* (capsule)														1		
***GARDEN PLANTS***																
*Coriandrum sativum*		1														
***WILD SPECIES***																
**Apiaceae**																
*Pimpinella* cf. *major*		1														
*Apiaceae*											1					
**Amaranthaceae**																
*Bassia hyssopifolia*		1														
*Atriplex* sp.																11
*Chenopodium* sp.	1	11		1	2		1				1		1			
Amaranthaceae		1														
**Asparagaceae**																
*Muscari* sp.				1												
**Asphodelaceae**																
*Asphodelus fistulosus*				1											1	
**Boraginaceae**																
Boraginaceae																1
**Brassicaceae**																
*Calepina irregularis*		1														
Brassicaceae	1															
**Caryophyllaceae**																
*Silene* sp.		4		2			1									1
*Spergularia arvensis*				2												
Caryophyllaceae													1			1
**Cyperaceae**																
*Carex* sp.		12		2		1							1	1		1
Cyperaceae		2														
**Fabaceae**																
*Medicago lupulina*		2														
*Medicago* sp.					2											
*Trifolium* sp.		1														
Fabaceae		2		1												
**Juglandaceae**																
*Juglans regia* frag.													1			
**Lamiaceae**																
Lamiaceae				1												
**Malvaceae**																
*Malva* sp.																1
**Poaceae**																
*Festuca pratensis* type													1			
*Festuca* sp.			2			1										
*Lolium perenne* type		23		8												1
*Lolium persicum* type				13												
*Lolium temulentum*				15												
*Poa* type	1															
*Lolium* sp.	6				2											
*Milium* sp.					1											
*Phalaris* sp.				3												
*Lolium* sp.																
Poaceae	29	6		13	2		1				2					5
Poaceae frag.				2												
**Polygonaceae**																
*Polygonum* sp.		3		5	1		2									
*Rumex* sp.				2	4											
**Rosaceae**																
*Prunus spinosa*		6														
*Rubus* sp.		38														
*Rubus idaeus*		34														
*Rubus idaeus/fruticosus*				3												
*Rubus* sp.	1															
*Rosa* sp.													2			
*Sorbus* sp.		1														
Rosaceae					2											
**Rubiaceae**																
*Asperula arvensis*																1
*Asperula* sp.											1					
*Galium* sp.	1	6		3			1					1	3			4
*Sherardia arvensis*					1											
Indeterminated	1	33		8	7		2						4	1		3

## Data Availability

Data are contained within the article.
